# Evaluation of the Effects of High Silver and Copper Nanoparticle Concentrations on *Vaccinium myrtillus* L. under Field Conditions

**DOI:** 10.3390/nano14191545

**Published:** 2024-09-24

**Authors:** Alexandra Peshkova, Inga Zinicovscaia, Liliana Cepoi, Ludmila Rudi, Tatiana Chiriac, Nikita Yushin, Larisa Ganea

**Affiliations:** 1 Joint Institute for Nuclear Research, 6 Joliot-Curie Str., 141980 Dubna, Russia; 2 Doctoral School Biological, Geonomic, Chemical and Technological Science, State University of Moldova, 60 Alexei Mateevici Str., MD-2009 Chisinau, Moldova; 3 Horia Hulubei National Institute for R&D in Physics and Nuclear Engineering, 30 Reactorului Str., 077125 Măgurele, Romania; 4 Institute of Microbiology and Biotechnology, Technical University of Moldova, 1 Academiei Str., MD-2028 Chisinau, Moldova

**Keywords:** copper nanoparticles, silver nanoparticles, bilberry, ICP-OES, particle-induced X-ray emission technique, biochemistry

## Abstract

The extensive development of nanotechnologies has allowed nanoparticles to impact living systems through different pathways. The effect of single exposure to high concentrations of silver and copper nanoparticles (50–200 mg/L) on *Vaccinium myrtillus* L. under field conditions was investigated. Nanoparticle uptake in different segments of *Vaccinium myrtillus* L. was assessed by applying inductively coupled plasma–atomic emission spectroscopy and a particle-induced X-ray emission technique. Copper nanoparticles mainly accumulated in the roots and leaves, while silver nanoparticles showed a higher affinity for the roots and berries. The nanoparticles’ effects on the pigments and antioxidant activity of the plant’s leaves were also evaluated. The possible human health risk associated with the consumption of nanoparticle-contaminated berries was assessed. The results indicated that the consumption of berries contaminated with nanoparticles presented a low risk for human health.

## 1. Introduction

The rapid development of nanotechnology and intensive nanoparticle production have raised serious concerns over the impact of nanoparticles on the environment and humans [1]. Based on their specific physical and chemical properties (size, shape, surface coating, and chemical composition), metal nanoparticles (NPs) are the most commonly used nanomaterials in medicine, agriculture, industry, and commercial products [1,2]. According to Inshakova and Inshakov [3], silver nanoparticles (AgNPs) are the most commercialized NPs, constituting more than 50% of consumer products containing NPs. Because of their antimicrobial, electrical, and optical properties, AgNPs are applied in medicine, optics, electronics, cotton fabrics, food packaging production, etc. [4,5,6]. Every year, the production of copper nanoparticles (CuNPs) increases; they are widely used in medicine, the production of polymeric materials and textiles, electronics, medicine, and food packaging [7,8,9,10,11,12,13]. It is expected that the market size value of their production will reach USD 4.5 billion by the end of 2030 [14].

In the agricultural sector, CuNPs and AgNPs can be used as nanofertilizers to enhance crop yields, as well as to control and avoid plant diseases [15,16,17,18]. As an example, Yan and co-authors [19] showed that treating seeds with AgNPs increased their resistance to biotic and abiotic stresses and facilitated germination. It should be noted that copper is an important micronutrient indispensable for plant growth that also plays a key role in increasing plant pathogen resistance. In comparison with traditional fertilizers, the size effect and slow metal ion release from metal NPs increase the soil uptake efficiency, thereby lowering the required application dose [20].

The versatility of metal NP usage inevitably leads to their release into the environment, mainly through wastewater, atmospheric emissions, solid household, and municipal waste, as well as intentional agricultural application [21,22,23,24,25,26,27]. According to Keller et al. [10], 63–91% of the produced NPs end up in landfills, 8–28% accumulate in the soil, 0.4–7% are released in water bodies, and 0.1–1.5% are emitted in the atmosphere. Klaine and co-authors [28] suggested that soil may become a major sink for CuNPs released into the environment as a result of industrial activity. The annual increase in the content of AgNPs in soils treated with sewage sludge varies from 662 to 1571 ng/kg for nano-Ag. In surface waters in the USA, the concentration of AgNPs is at the level of 0.116 ng/L, and in Europe, it is 0.764 ng/L [21]. Of the 2500 tons of AgNPs produced in the USA per year, around 150 tons are deposited in sludge, and 80 tons are released in surface waters [29].

Plants that interact with objects in the environment are subjected to metal NP exposure. Since plants are widely consumed by humans, their NP content may have negative impacts on the population [30]. The effect of NPs on plants depends on the plant species, growth conditions, and type of NPs, as well as their concentration, chemical form, coating, and size [30,31]. Although a considerable amount of research on the effect of metal NPs on plants has been performed, the majority was carried out in hydroponics and pots, or at low NP concentrations, and usually under controlled conditions [30,32,33,34].

The bilberry (*Vaccinium myrtillus* L.) is a perennial shrub of the Ericaceae family, native to Northern Europe and North America [35]. Bilberry berries are highly regarded in many countries for their pleasant taste; they are often used to make jams, preserves, pies, juices, and alcoholic beverages. Among berries, bilberry exhibits the highest antioxidant properties. Its fruit and leaf extracts are used for the treatment of eye and cardiovascular diseases and the regulation of high sugar levels in the blood [36,37,38,39].

To our knowledge, the effect of high concentrations of metal NPs on plants in a field experiment has not been investigated. The aim of the present study was to assess the effects of a single exposure to high AgNP and CuNP concentrations on *Vaccinium myrtillus* L., which is widely used for nutrition and medicinal purposes. To achieve this goal, the following objectives were fulfilled: (1) to quantize the uptake of silver and copper in bilberry segments; (2) to investigate the effects of the NPs on the plant’s biochemical composition and antioxidant activity; and (3) to evaluate the health risk associated with the consumption of contaminated berries.

## 2. Materials and Methods

### 2.1. Nanoparticles and Plant Material

Copper and silver nanoparticles (200 mg of metal/L) were purchased from M9 (Tolyatti, Russia). The chemically produced silver and copper nanoparticles were stabilized by a cationic polymer (polyvinylpyrrolidone). The nanoparticles were completely soluble in water and alcohols. For the experiment, initial solutions were diluted to obtain solutions with concentrations of 50 mg/L and 100 mg/L. Mature bilberry plants were used as the object of study.

### 2.2. Experiment Design

For the field experiment, a mixed forest area was selected (Dubna, Russia). AgNP and CuNP solutions with concentrations of 50, 100, and 200 mg/L were used for single-time watering of bilberry bushes at the flowering stage. Each type of NP in a volume of 500 mL was used to water plants growing on an area of 1 m^2^; the distance between experimental plots was 2 m. The experiment lasted for 60 days, starting from the flowering period to the fruiting stage. Three plants were used for each type of nanoparticle solution. At the end of the experiment, berries, leaves, stems, roots, and soil were collected from each plant. Prior to analysis, the roots were washed with distilled water to remove soil particles. The samples for elemental analysis were dried at 50 °C, and for biochemical analysis, they were frozen at −80 °C.

### 2.3. Analytical Techniques

A Thermo Scientific Talos F200i (Thermo Fisher Scientific, Waltham, MA, USA) transmission electron microscope (TEM) was used to characterize metal NPs. An inductively coupled plasma–optical emission spectrometer (ICP-OES) PlasmaQuant PQ 9000 Elite (Analytik Jena, Jena, Germany) was used to determine the copper content. Sample preparation was performed according to the procedure described in [39]. A particle-induced X-ray emission (PIXE) technique at the 3 MV Tandetron (IFIN-HH, Magurele, Romania) was applied to detect and quantify the silver content in the samples. Details about the sample analysis are presented in [40]. Analysis of reference materials INCT PVTL-5 (Oriental Basma tobacco leaves), NIST 1573a (tomato leaves), and NIST 2709a (San Joaquin Soil Baseline Trace Element Concentrations) ensured the quality of the measurements; the difference between the certified and obtained values was <5%.

### 2.4. Biochemical Analysis and Antioxidant Activity

#### 2.4.1. Determination of Pigment Content

Chlorophyll and carotenoids were extracted from the macerated leaf biomass using 80% acetone at room temperature by shaking for 12 h. The obtained suspension was centrifuged for 7 min at 4000× *g*. The absorbance was recorded at 470 nm, 668.2 nm, and 646.8 nm. The content of carotenoids and chlorophyll was determined spectrophotometrically with calculations based on specific coefficients, as presented in [41].

#### 2.4.2. Determination of Antioxidant Activity

First, 5 mL of 55% ethyl alcohol solution was added to 500 mg of dry biological material. Then, the samples were macerated for 2 h under continuous agitation. The extracts were then separated from the biological material by centrifugation and filtration. The obtained extracts were stored at 0 °C. The ABTS radical (2,2′-azino-bis (3-ethylbenzothiazoline-6-sulfonic acid) was prepared by mixing ABTS (7 mM concentration) with 2.45 mM potassium persulfate. Next, the ABTS working solution was adjusted to an absorbance of 0.700 ± 0.02 at 734 nm. The reaction mixture consisted of 2.7 mL of ABTS solution and 0.3 mL of the sample. The process of ABTS reduction lasted 6 min. The antioxidant activity was evaluated by the percentage inhibition of ABTS [42].

The DPPH radical (2,2-diphenyl-1-picrylhydrazyl) was prepared to form a solution of 60 µM DPPH in 96% ethanol. The reaction mixture contained 2.7 mL of DPPH solution and 0.3 mL of the hydro-ethanol extract from the biological material. The samples were incubated at room temperature in the dark for 30 min and then the absorbance was recorded at 517 nm. The antioxidant activity was evaluated by the percentage inhibition of DPPH [43].

#### 2.4.3. Determination of the Phenolic Content

Folin–Ciocalteu reagent in a 1:9 dilution was applied. The working mixture consisted of 1.5 mL Folin–Ciocalteu reagent, 1.2 mL 7.5% sodium bicarbonate solution, and 0.3 mL of the sample. After an incubation period of 5 min at 50 °C, the absorbance was measured at 760 nm. The phenolic compound content was calculated based on the calibration curve for gallic acid [44].

### 2.5. Translocation and Bioconcentration Factors

The translocation factors (*TFs*) and bioconcentration factors (*BCFs*) were calculated to assess the uptake of NPs from the soil environment and their transfer in the root–stem–leaf–berry system [45].

(1) $$BCF=C_{R\;\left(St,\;L\;or\;B\right)}/C_{S\;}$$

(2) $$TF=C_{St\;\left(\;L\;or\;B\right)}/C_{R\;};\;\;TF=C_{L\;\left(B\right)}/C_{St\;};\;\;TF=C_{B}/C_{L\;}$$

where *C_R_*—element concentration in roots, mg/kg; *C_St_*—element concentration in stems, mg/kg; *C_L_*—element concentration in leaves, mg/kg; *C_B_*—element concentration in berries, mg/kg; and *C_S_*—element concentration in soil, mg/kg.

### 2.6. Human Health Risk

The estimated daily intake (EDI, mg/kg bw day) of elements was calculated according to Equation (3):

(3) $$EDI=\frac{C_{i}\times IR\times EF\times ED}{AT\times BW}$$

where *Ci* is the metal content in berries, mg/kg; *IR* is the berry ingestion rate, kg; *BW* is the average human body weight, 70 kg; *EF* is the exposure frequency, 365 days/year; *ED* is the exposure duration, 70 years; and *AT* is the average exposure time, *EF* × *ED* [46,47]. A conversion factor of 0.17 for bilberry was used to convert dry weight to fresh weight [48].

In order to evaluate the non-carcinogenic effects of a metal on human health through the consumption of berries, the hazard quotient (*HQ*) was computed (Equation (4)):

(4) $$HQ=\frac{EDI}{RfD}$$

where *RfD* is the daily oral reference dose. In this study, 0.04 mg·kg/day was used for copper [49] and 0.005 mg·kg/day was used for silver [50].

### 2.7. Statistical Analysis

All measurements were performed in three replicates. The data were statistically analyzed by calculating the averages, standard deviations, and confidence intervals using Student’s *t*-test. The data were considered statistically significant at a *p*-value ≤ 0.05. Microsoft Excel 2021 and Statistica 10 were used for statistical analysis.

## 3. Results and Discussion

**Figure 1:**
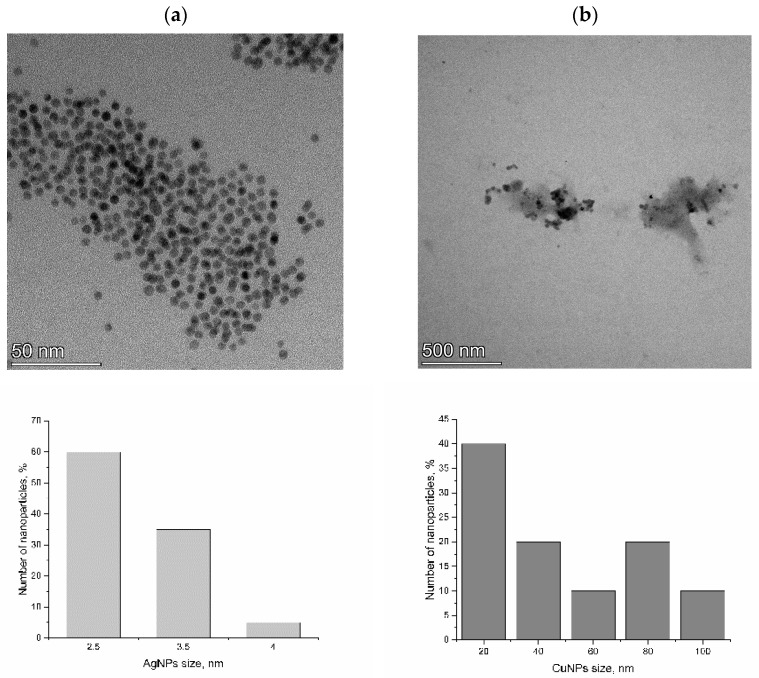
TEM images of (**a**) silver and (**b**) copper nanoparticles, as well as their size distributions.

According to the TEM images (Figure 1), both the silver and copper nanoparticles had a spherical shape. The diameter of CuNPs varied from 20 to 100 nm, and that of AgNPs was in the range of 2.5–4 nm.

### 3.1. Copper Uptake in Vaccinium myrtillus L.

**Figure 2:**
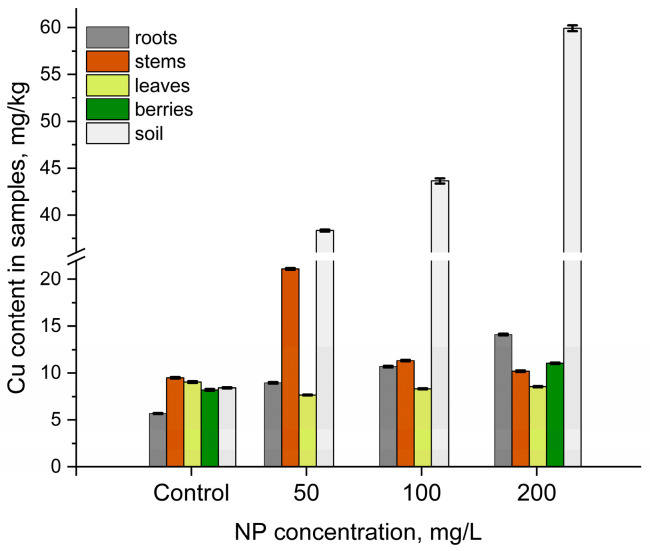
Content of copper in segments of *Vaccinium myrtillus* L. watered with CuNPs at concentrations of 50–200 mg/L under field conditions. Error bars represent the standard deviation of the measurements.

Copper is a crucial micronutrient needed for plant growth, and the typical level in soils is 5–30 mg/kg. According to Ballabio et al. [51], the average content of copper in the soils of broadleaf forests is 17.66 mg/kg, almost twice as high as in coniferous forests (9.37 mg/kg). In the control, the bilberry sample content of copper was 5.67, 9.49, 9.03, 9.42, and 8.2 mg/kg in the roots, stems, leaves, soil, and berries, respectively (Figure 2). According to Klavins et al. [38], the content of copper in bilberries collected in different countries ranged from 1.54 mg/kg (Spain) to 8.91 mg/kg (Latvia).

The content of copper in the soil watered with NPs increased in a dose-dependent manner, and the maximum value of 59.92 mg/kg was obtained when 200 mg/L of CuNPs were used. The same pattern was characteristic for the roots, where the content of copper increased from 8.9 to 14.1 mg/kg and was 1.6–2.5 times higher than in the control samples. There was a clear correlation found between the copper content in the soil and roots (r = 0.97 at *p* < 0.005).

The level of copper in the leaves of the sampled plants was lower than in the control (*p* < 0.005), which is in agreement with previously published studies. Kohatsu et al. [52] studied the effect of copper-based nanoparticles and CuSO_4_ on lettuce plants and showed that none of the copper forms introduced into the soil accumulated in the leaves [52]. Xiao et al. [53] found that the content of copper in the roots of soybeans grown in the presence of CuNPs (10–30 nm) was significantly higher than in other parts of the plants. The authors also emphasized that the copper content was higher in plants treated with NPs compared with plants treated with copper salts [53]. Dose-dependent accumulation of copper in the root system of *Petroselinum crispum* and low translocation to leaves was reported in [54]. Copper accumulation in root tissues and minimal translocation to aboveground parts of plants was also shown in [55]. Berries collected from plants watered with CuNPs at a concentration of 200 mg/L contained 34% more copper than the berries from control plants. In the present study, bilberry watered with CuNPs at concentrations of 50 and 100 mg/L did not produce berries. CuNPs are known to have a negative effect on plant metabolism. For example, in *Arabidopsis thaliana*, CuNPs decreased the sugar substance and increased lipid peroxidation, which plays a significant role in plant yield and also affects the hindrance to root development and plant biomass [56]. The application of CuNPs resulted in a decrease in the total aerial biomass of strawberries, as well as the number of fruits and fruit weight [57].

The values of the BCF and TF (Table 1) showed low copper uptake from soil in plants (BCF < 1) and translocation among plant segments. TF values approximately equal to or higher than one were obtained mainly for control plants, as well as for the stem/root system at a CuNP concentration of 50–100 mg/L and the berry–stem–leaf system at an NP concentration of 200 mg/L.

This is in agreement with our previous study, in which it was shown that the BCF values for CuNPs in parsley segments were less than one [54]. Yu and co-authors [58] showed that the BCF for CuNPs in species in the aquatic food chain was lower than the BCF values reported for Cu ions.

### 3.2. Silver Uptake in Vaccinium myrtillus L.

**Figure 3:**
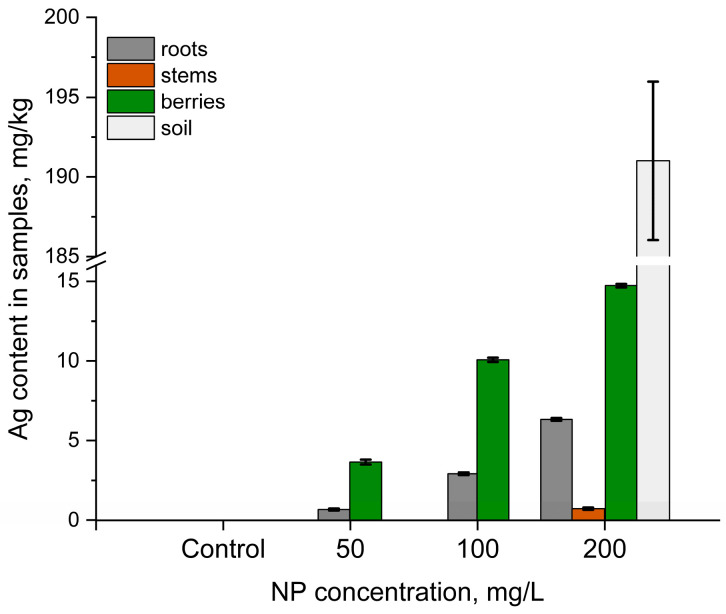
Content of silver in segments of *Vaccinium myrtillus* L. watered with AgNPs at concentrations of 50–200 mg/L under field conditions. The error bars represent the standard deviation of the measurements.

The average silver content in the soils varied between 0.03 and 0.09 mg/kg, while in plant ash, it varied from 0.03 to 2 mg/kg of dry biomass [54]. The content of silver in the control soil and the soil subjected to 50 and 100 mg/L of AgNPs was below the PIXE detection limit (30 mg/kg). In the soil samples exposed to 200 mg/L AgNPs, it was on the level of 191 mg/kg (Figure 3).

Although silver was not detected in the roots of the control plants, its content in the sampled plants increased from 0.7 (50 mg/L) to 6.3 (200 mg/L) mg/kg. The silver content in the leaves and stems was below the PIXE detection limit, except for the stems of plants treated with 200 mg/L of AgNPs. In the berries, as in the roots, the silver content increased proportionally to the increase in AgNP concentration. Since silver was not detected in most of the soil samples or plant segments, BCF values were calculated only for the samples exposed to 200 mg/L of AgNPs. The BCF value was less than one (Table 1). In the case of TF, the active uptake of silver in berries from roots was observed.

The translocation of AgNPs in plant tissues was reported in other studies as well, and it depends on the particle size, plant species, and conditions of nanoparticle application. It has been noted that small AgNPs can pass through pores, unlike larger particles. However, under the impact of AgNPs, large pores can be formed, ensuring the penetration of larger particles through the cell wall [34,59]. Geisler-Lee et al. [60] showed the uptake of AgNPs with a size of 20 nm by *Arabidopsis thaliana* roots and their transport throughout the plant [60].

### 3.3. Effects of Nanoparticles on Vaccinium myrtillus L. Leaf Biochemical Composition and Antioxidant Activity

**Figure 4:**
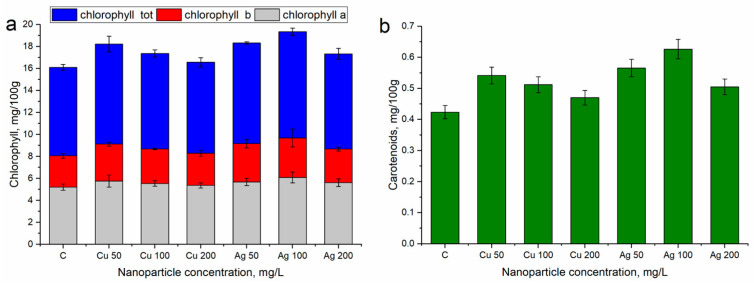
Pigment content of (**a**) chlorophyll and (**b**) carotenoids in bilberry leaves watered with nanoparticles at concentrations of 50–200 mg/L under field conditions. The error bars represent the standard deviation of the measurements.

Figure 4 presents the content of pigment in the leaves of the control plants and those watered with NPs. Both types of NPs applied to the bilberry plants induced a significant increase in the carotenoid content, while the content of chlorophyll either increased or remained at the level in the control samples.

At a CuNP concentration of 50 mg/L, the total chlorophyll content exceeded the control value by 13.1% (*p* ≤ 0.1). The increase was more pronounced for chlorophyll b, the content of which exceeded the control level by 18.0% (*p* ≤ 0.05), while the content of carotenoids increased by 27.7% (*p* ≤ 0.01). At a CuNP concentration of 100 mg/L, the increasing trend in the chlorophyll content was maintained; yet, only the content of chlorophyll b was significantly higher than in the control (by 10.7%, *p* ≤ 0.05), and the carotenoid content increased by 20.8% (*p* ≤ 0.01). At a CuNP nanoparticle concentration of 200 mg/L, the level of chlorophyll was similar to the control, while carotenoids surpassed it by 10.9%; however, this result was not statistically significant (*p* = 0.18).

For the three applied AgNP concentrations, the total chlorophyll content significantly increased compared with the control (by 17.5–20.0%, *p* < 0.05). The carotenoid content in the sampled leaves was also 19.1–47.7% (*p* < 0.05) higher. The highest increase in the pigment content compared with the control was observed at an AgNP concentration of 100 mg/L (by 47.7%, *p* < 0.001).

**Figure 5:**
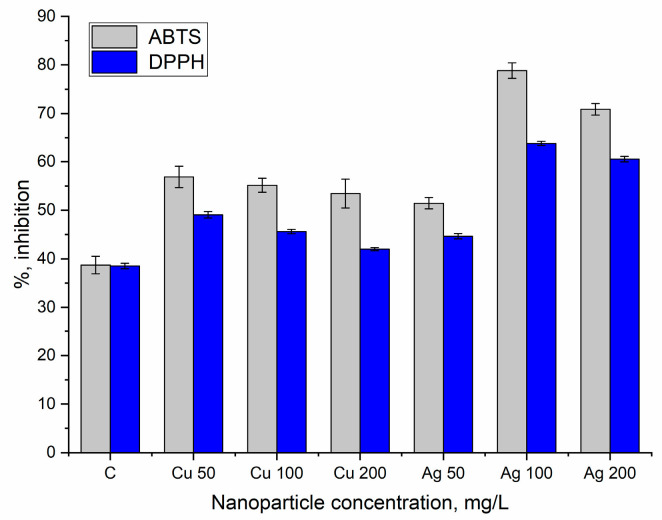
Radical-scavenging capacity of extracts from bilberry leaves watered with nanoparticles at concentrations of 50–200 mg/L under field conditions. The error bars represent the standard deviation of the measurements.

The radical-scavenging capacity of extracts from bilberry leaves treated with CuNPs and AgNPs against DPPH and ABTS radicals (Figure 5) significantly exceeded the control. For CuNPs, at all applied concentrations, the radical-scavenging capacity against the ABTS radical cation showed very similar values in the range of 38.0–46.9% (*p* ≤ 0.001) compared with the control. An increase in the activity of extracts from the leaves of plants watered with 50 and 100 mg/L NPs against the DPPH radical was observed (by 27.4 and 18.3%, respectively). In the case of AgNPs, the radical-scavenging capacity increased at all applied NPs concentrations (by 15.9–65.7% against DPPH (*p* < 0.001) and by 32.9–103.7% against the ABTS radical (*p* < 0.001)). The highest values of radical-scavenging activity were attained for NP concentrations of 100 and 200 mg/L.

**Figure 6:**
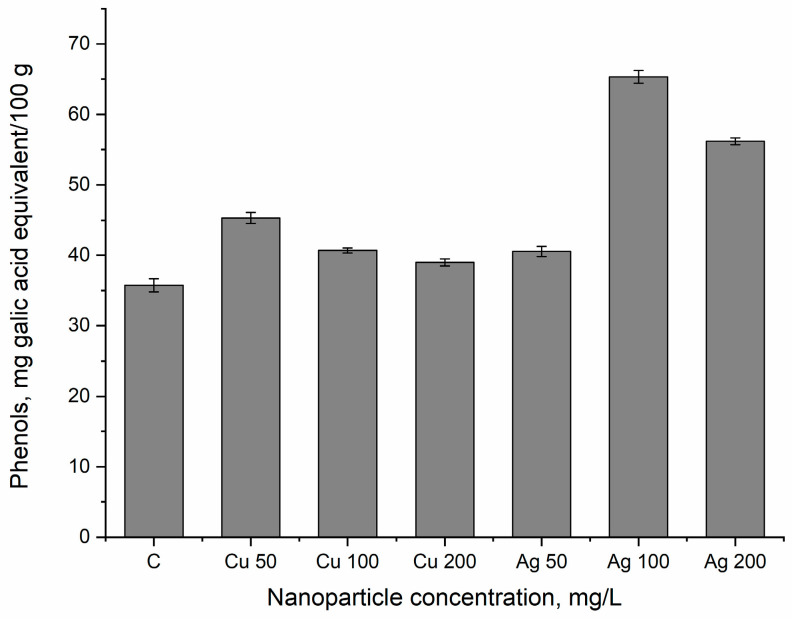
The content of phenols in bilberry leaves watered with nanoparticles at concentrations of 50–200 mg/L under field conditions. The error bars represent the standard deviation of the measurements.

The change in the content of phenols in the plants subjected to NPs followed the same pattern as the radical-scavenging activity (Figure 6). For CuNPs, the highest value was obtained at a concentration of 50 mg/L, at which the content of phenol increased by 26.8% compared with the control (*p* ≤ 0.001). The AgNP concentrations of 100 and 200 mg/L increased the phenol content by 82.7 and 57.1%, respectively, in comparison with the control (*p* ≤ 0.001).

The impact of metallic nanoparticles on the plant physiological, biochemical, and agronomic parameters has been investigated in many species in different studies. However, the reported results vary greatly and are sometimes contradictory. For example, high concentrations of CuNPs did not affect the growth or pigment content in *Origanum vulgare* [61]. In contrast, CuNPs at a concentration of 38 mg/L reduced the chlorophyll content in *Elodea canadensis* [62], while a lower concentration of 1.0 mg/L had similar effects on *Landoltia punctata* [63]. The treatment of *Triticum aestivum* plants for six days with CuNP solutions with sizes below 50 nm and concentrations of 1, 5, 10, and 50 ppm resulted in a reduction in chlorophyll content; the negative impact was associated with a decrease in the content of proteins involved in photosynthesis and tetrapyrrole synthesis [64]. Additionally, it was shown that CuNPs at concentrations of 69.4 µM enhanced drought tolerance in maize, helping to maintain chlorophyll and carotenoid content under drought conditions [65]. Copper and AgNPs did not change the level of total chlorophyll in amaranth and wheat, but they resulted in significant changes in chlorophyll contents in rice and maize [66].

The impact of AgNPs on plant physiology also varies depending on species and treatment conditions. Kale seeds treated with AgNPs at a concentration of 50 mg/L showed an increase in chlorophyll content, alongside a reduction in carotenoid and phenol levels in leaves. Conversely, foliar treatment with AgNPs at concentrations of 20, 40, and 60 mg/L enhanced the levels of chlorophyll, carotenoids, and phenols in *Trigonella foenumgraecum* plants, with a concentration-dependent stimulatory effect [67].

The application of AgNPs at a range of concentrations (0, 25, 50, 100, 200, and 400 ppm) improved the antioxidant status of 7-day-old *Brassica juncea* seedlings, indicating potential oxidative stress mitigation [68]. In a study on *Solanum melongena* L., treatment with 0.1 µmol of AgNPs under drought conditions led to an 80% increase in photosynthetic pigments, including chlorophyll a, chlorophyll b, and carotenoids, underscoring the ability of AgNPs to support photosynthetic function and reduce the impact of drought stress on plants [69]. Furthermore, in research conducted by Dziwulska-Hunek [70], leaves of *Cucurbita pepo* L. grown from seeds treated with AgNPs exposed to laser light and alternating magnetic fields exhibited significant increases in chlorophyll a and b content (53% and 11%, respectively) and carotenoids (79%). Similarly, in leaves from potted plants, there was an increase in chlorophyll a content (42–43%) and chlorophyll b (25%), with carotenoid levels increasing by 66% to 81%, demonstrating the positive impact of AgNPs on the biochemical composition and antioxidant status of plants.

These findings suggest that AgNPs and CuNPs may offer a promising method for enhancing plant health and productivity in modern agriculture. However, concentration and application methods require careful consideration to optimize benefits and minimize potential adverse effects.

### 3.4. Human Health Risk Assessment

According to the World Health Organization, a person needs to consume more than 400 g of fruits and vegetables per day five portions, 80 g per portion) [71,72]. In the present study, it was considered that berries constitute one-fifth (80 g) of the total fruit and vegetable consumption. Thus, to calculate EDI and HQ values, ingestion rates of 80 g were taken into account (Table 2).

According to the obtained results, the EDI values were less than the daily oral reference dose, which indicated no risk to human health. The high concentration of silver or copper in the berries resulted in high EDI values. These results were supported by HQ values. For bilberries contaminated with NPs, the HQ values were less than one, indicating low adverse health effects (Table 2).

## 4. Conclusions

A field experiment involving a single-dose exposure of *Vaccinium myrtillus* to high concentrations (up to 200 mg/L) of AgNPs and CuNPs allowed for the assessment of copper and silver distribution across plant segments. The maximum content of copper was determined in the soil (59.92 mg/kg) and root system (14.11 mg/kg), with minimal translocation to the leaves and berries. In contrast, exposure to AgNPs resulted in significant silver accumulation in the berries via the root system, while the silver levels in the stems and leaves remained below the detection limits of PIXE. The maximum silver content of 14.72 mg/kg was detected in bilberries exposed to 200 mg/L of AgNPs. Both types of NPs induced an increase in the pigment content and antioxidant activity. The effect was more pronounced in the case of AgNPs. Although the hazard quotient values indicated low human health risks associated with the consumption of berries contaminated with AgNPs and CuNPs, further studies encompassing a broader range of NP concentrations and sizes need to be performed.

## Figures and Tables

**Table 1:** BCF and TF for bilberry plant segments subjected to a single exposure to CuNP and AgNP solutions.

		BCF				TF					
		Roots	Stems	Leaves	Berries	Stems/Roots	Leaves/Roots	Berries/Roots	Leaves/Stems	Berries/Stems	Berries/Leaves
CuNPs	0	0.67	1.13	1.07	0.97	1.67	1.59	1.45	0.95	0.86	0.91
	50	0.23	0.55	0.20	-	2.36	0.86	-	0.36	-	-
	100	0.24	0.26	0.19	-	1.06	0.78	-	0.79	-	-
	200	0.24	0.17	0.14	0.18	0.72	0.61	0.78	0.84	1.08	1.29
AgNPs	0	-	-	-	-	-	-	-	-	-	-
	50	-	-	-	-	-	-	5.32	-	-	-
	100	-	-	-	-	-	-	3.46	-	-	-
	200	0.03	0.004	-	0.08	0.12	-	2.32	-	19.84	-

**Table 2:** EDI and HQ values for bilberry collected from plants watered with AgNPs and CuNPs.

NP Concentration, mg/L		EDI	HQ	Content in Berries, mg/kg
Cu	Control	1.59 × 10^−3^	3.99 × 10^−2^	8.21
	200	2.14 × 10^−3^	5.35 × 10^−2^	11.01
Ag	50	7.13 × 10^−4^	1.43 × 10^−1^	3.67
	100	1.96 × 10^−3^	3.91 × 10^−1^	10.07
	200	2.86 × 10^−3^	5.72 × 10^−1^	14.72

## Data Availability

The data presented in this study are available on request from the corresponding author.
